# Identification of theranostic factors for patients developing metastasis after surgery for early-stage lung adenocarcinoma

**DOI:** 10.7150/thno.53176

**Published:** 2021-01-26

**Authors:** Wei-Chung Cheng, Chun-Yu Chang, Chia-Chien Lo, Chih-Ying Hsieh, Ting-Ting Kuo, Guan-Chin Tseng, Sze-Ching Wong, Shu-Fen Chiang, Kevin Chih-Yang Huang, Liang-Chuan Lai, Tzu-Pin Lu, K.S. Clifford Chao, Yuh-Pyng Sher

**Affiliations:** 1 Ph.D. Program for Cancer Biology and Drug Discovery, China Medical University and Academia Sinica, Taiwan.; 2 Graduate Institute of Biomedical Sciences, China Medical University, Taichung 404, Taiwan.; 3 Research Center for Cancer Biology, China Medical University Hospital, Taichung 404, Taiwan.; 4 Center for Molecular Medicine, China Medical University Hospital, Taichung 404, Taiwan.; 5 Department of Anatomic Pathology, Nantou Hospital of the Ministry of Health and Welfare, Nantou 540, Taiwan.; 6 Lab of Precision Medicine, Feng-Yuan Hospital, Ministry of Health and Welfare, Taichung 420, Taiwan.; 7 Translation Research Core, China Medical University Hospital, Taichung 404, Taiwan.; 8 Department of Biomedical Imaging and Radiological Science, China Medical University, Taichung 404, Taiwan.; 9 Graduate Institute of Physiology, College of Medicine, National Taiwan University, Taipei 100, Taiwan.; 10 Department of Public Health, National Taiwan University, Taipei 100, Taiwan.; 11 Cancer Center, China Medical University Hospital, Taichung 404, Taiwan.; 12 School of Medicine, China Medical University, Taichung 404, Taiwan.; 13 Chinese Medicine Research Center, China Medical University, Taichung 404, Taiwan.; 14 Research Center for Chinese Herbal Medicine, China Medical University, Taichung 404, Taiwan.

**Keywords:** Lung adenocarcinoma, prognostic biomarkers, early-stage lung adenocarcinoma.

## Abstract

**Rationale:** Lung adenocarcinoma (LUAD) is an aggressive disease with high propensity of metastasis. Among patients with early-stage disease, more than 30% of them may relapse or develop metastasis. There is an unmet medical need to stratify patients with early-stage LUAD according to their risk of relapse/metastasis to guide preventive or therapeutic approaches. In this study, we identified 4 genes that can serve both therapeutic and diagnostic (theranostic) purposes.

**Methods:** Three independent datasets (GEO, TCGA, and KMPlotter) were used to evaluate gene expression profile of patients with LUAD by unbiased screening approach. Upon significant genes uncovered, functional enrichment analysis was carried out. The predictive power of their expression on patient prognosis were evaluated. Once confirmed their theranostic roles by integrated bioinformatics, we further conducted *in vitro* and *in vivo* validation.

**Results:** We found that four genes (*ADAM9*, *MTHFD2*, *RRM2,* and *SLC2A1)* were associated with poor patient outcomes with an increased hazard ratio in LUAD. Knockdown of them, both separately and simultaneously, suppressed lung cancer cell proliferation and migration ability *in vitro* and prolonged survival time in metastatic tumor mouse models. Moreover, these four biomarkers were found to be overexpressed in tumor tissues from LUAD patients, and the total immunohistochemical staining scores correlated with poor prognosis.

**Conclusions:** These results suggest that these four identified genes could be theranostic biomarkers for stratifying high-risk patients who develop relapse/metastasis in early-stage LUAD. Developing therapeutic approaches for the four biomarkers may benefit early-stage LUAD patients after surgery.

## Introduction

Lung cancer is a complex and aggressive disease. About 80% of lung cancer is non-small cell lung cancer (NSCLC), of which lung adenocarcinoma (LUAD) is the most common subtype. Most LUAD patients die of aggressive metastatic disease, with an average 5-year survival of 17.1% worldwide 1. About 30% of LUAD patients are diagnosed at an early stage with limited disease symptoms and are treated with surgical resection plus adjuvant therapy according to various protocols, with the intent to cure 2. Nevertheless, large percentages (20-50%) of these patients eventually have a relapse of LUAD because of undetected residual disease or metastases 3. For LUAD patients diagnosed with advanced stage III and IV or metastatic disease, treatment options are limited, the prognosis is poor, and survival rates are low.

Owing to the development of screening techniques such as low-dose computed tomography, a significantly increasing number of lung cancer patients are diagnosed at early stages 4. According to the newest National Comprehensive Cancer Network (NCCN) Guidelines (version 5.2018), the most widely recognized standard for clinical policy in oncology, early-stage NSCLC patients (stage I and II) should undergo surgical exploration and resection if they are operable 5. If the tumor margins are negative, stage IA patients do not require adjuvant treatment, but high-risk patients at stage IB and IIA are suggested to undergo adjuvant chemotherapy. However, the definition of 'high-risk patients' is still challenging. Moreover, some stage IA patients, who will not receive adjuvant treatment according to NCCN Guidelines, still will be diagnosed with relapse within five years. Based on these observed situations, improved diagnosis and therapeutic advice for early-stage patients should be considered an imperative obligation.

Inhibitors or antibodies of NSCLC-driver receptors have been among the most successful examples of targeted cancer therapies to date. It is estimated that only 15-25% of NSCLC patients benefit from immunotherapy, suggesting there is a need to explore additional novel biomarkers as potential targets 6. Due to the heterogeneity of mutated genes causing cancer in the individual patient, precision medicine, which considers this individual variability and uses different prevention and treatment strategies to benefit each patient, has become critical for cancer treatment 7. Therefore, there is an urgent clinical need for new biomarkers that can help with early-stage diagnosis, improve prognostication, and predict response to various therapies, enabling more individualized patient treatment. Unfortunately, there is no reliable predictive biomarker available in LUAD to identify this high-risk population for therapy intensification.

Theranostic biomarkers have both therapeutic and diagnostic functions in monitoring early response to treatment and predicting treatment efficacy 8. Some biomarkers may be more appropriately defined as theranostic markers, if they have the ability to indicate targeted therapy based on a specific diagnostic test, such as driver oncogenes (EGFR/ALK/ROS1/BRAF) and biomarkers for immunotherapy with PDL1 9. Although many cancer theranostics have been reported, the therapeutic strategies for LUAD have room for improvement via properly validated biomarkers for prognostic features. It is essential to continue to look for more effective treatments, targeting precisely the biological characteristics of each tumor. Due to the limited effects of targeting a single pathway, ongoing trials are focusing on combination regimens to target multiple pathways for improved anti-tumor efficacy.

To achieve the goals of personalized precision medicine, we need to develop robust methods for characterizing and stratifying patients, including computational tools for analyzing large datasets, rigorous testing techniques, and evidence-based clinical practices. In this study, we have identified and validated 4 prognostic biomarkers, which may serve as useful tools for stratifying early-stage LUAD patients according to their risk of relapse. The 4 biomarkers contribute to mechanisms that promote disease progression, including cancer cell growth and metastasis. The total immunohistochemical staining scores of the biomarkers were correlated with stage and poor survival time for early-stage LUAD patients. These findings suggest that targeting these four prognostic biomarkers may be a potential approach in lung cancer treatment.

## Material and Methods

### Bioinformatics analysis with the public LUAD datasets

Three independent datasets were used for evaluating the gene expression status of LUAD patients, GSE30219 and GSE31210 in the GEO database 10 and the LUAD dataset of TCGA obtained from our previous studies 11-13. Survival analysis was performed for the expression of genes of interest in the GEO datasets (GSE30219, GSE31210, GSE42127, and GSE 8894), the TCGA LUAD dataset, and the KMPlotter database (http://kmplot.com/analysis/) 14. The normalized RNA-seq data from TCGA was obtained from our published database, DriverDB 13, and integrating TCGA multi-omics data was from The Cancer Genomics Hub (CGHub, https://cghub.ucsc.edu). For the four microarray datasets used in this study, the normalized expression values were retrieved from the GEO database.

Functional enrichment analysis of the significant genes was also performed as detailed in our previous publications 11-13. In brief, for Geno Ontology, we used the topGO packages of R language to calculate the topology of the GO graph. In Pathway analysis, we used collections from KEGG 15, REACTOME 16, and MSigDB 17 to annotate the significant genes. The function/pathway with the adjusted p-value <0.05 were select for further analysis.

### Identification of the four prognostic biomarkers in lung adenocarcinoma

To increase the reliability of our method, we utilized several databases to screen potential biomarkers and took the results that were consistent across all of them. First, we identified 80 genes overexpressed in LUAD in three datasets (GSE30219, GSE31210, and the LUAD dataset of TCGA), containing 85, 226, and 513 tumor samples as well as 14, 20, and 59 adjacent normal tissues, respectively. Then, we manually selected 10 out of 80 biomarker candidates with high staining in lung tumors but no or low detection in normal lung tissues according to the IHC staining in the Human Protein Atlas (HPA) database, which is a resource for pathology-based biomedical research. Next, for the 10 candidate genes, we performed survival analysis in LUAD datasets from KMPlotter, as well as the two GEO datasets (GSE31210 and GSE30219) and the LUAD dataset from TCGA, to test whether their expression levels were related to survival. Finally, the 4 biomarkers, *ADAM9*, *MTHFD2*, *RRM2*, and *SLC2A1*, are selected for further analysis.

### Cell culture

The human lung adenocarcinoma cell lines A549 and CL1-0 and human embryonic kidney cell line 293T were obtained from the Bioresource Collection and Research Center (Hsinchu, Taiwan). A549 and CL1-0 cells were maintained in RPMI 1640 medium (Gibco, Australia), and 293T cells were maintained in Dulbecco's modified Eagle's medium (DMEM; Gibco) with 10% fetal bovine serum (FBS; Gibco) supplemented with 1% penicillin/streptomycin solution (Gibco). Lung cancer brain-metastatic subline Bm7 cells which were derived from CL1-0 were cultured in DME/F12 plus 10% FBS media as previously described 18. All of the cells were cultured in a humidified incubator containing 5% CO_2_ at 37 °C and were monitored periodically for mycoplasma free. Experiments were carried out within six months of acquiring the cells from the established cell bank to ensure that cells maintained their ability to form lung cancer tumors in SCID mice.

### Cell transfection and generation of gene knockdown clones

Four individual short hairpin RNA plasmids (sh*ADAM9*, sh*RRM2*, sh*MTHFD2,* and sh*SLC2A1*) and the negative control (sh*VOID*) were obtained from the National RNAi Core (Academia Sinica, Taipei, Taiwan) (Supplementary Table 1). The 293T cells were transfected with the control and shRNA plasmids using Lipofectamine 2000 reagent (Invitrogen, Carlsbad, CA, USA) to generate viral soups as we previously described 19. Knockdown of the target genes (*ADAM9, RRM2, MTHFD2*, and *SLC2A1)* in lung cancer cells was performed as previously described 20. Briefly, an equal amount of viral soups of individual shRNA (one volume) mixed with control sh*VOID* (3 volumes) or a combination of 4 individual shRNA mixtures (one volume each) were used to knock down the specific gene expression. The efficiency of knockdown was examined by immunoblotting. The stable knockdown clones of A549 and CL1-0 cells were prepared for *in vitro* functional assays. The stable 4 gene knockdown clones of A549 cells were prepared for* in vivo* studies.

### Immunoblotting

Total cell protein was extracted using RIPA lysis buffer. Protein concentrations were measured using the BCA protein assay (Bio-Rad, Hercules, CA, USA). The proteins were separated with 10% SDS-PAGE and were then transferred to PVDF membranes (Bio-Rad). Immunoblotting was performed by using primary antibodies overnight at 4 °C against ADAM9 (1:1000, GTX30025, GeneTex, CA, USA), RRM2 (1:1000, HPA056994, Sigma, MO, USA), MTHFD2 (1:1000, H00010797-M01, Abnova, Taiwan), SLC2A1 (1:1000, 07-1401, Millipore, Billerica, MA, USA), GAPDH (1:3000, 10494-1-AP, Proteintech, CA, USA) and α-Tubulin (1:3000, NB100-690, Novus, CO, USA), followed by incubation with horseradish peroxidase-conjugated secondary antibodies against mouse or rabbit IgG (1:10000, Santa Cruz, Dallas, TX, USA) for one hour at room temperature. GAPDH or α-tubulin served as the internal control. The blots were developed using an enhanced chemiluminescence assay (ECL; Millipore). The luminescent signals were assessed using the ChemiDoc^TM^ XRS+ image system and the associated Image Lab^TM^ software version 3.0 (Bio-Rad). Immunoreactive bands were analyzed and quantified using Quantity One version 4.6.8 (Bio-Rad).

### Colony formation assay

Cells were seeded and cultured in a six-well plate for 10 to 14 days (until visible colonies formed) with selective medium replacement every three days. At the end of the experiment, colonies were fixed with 50% methanol, and then the cells were stained with 0.5% crystal violet at room temperature for 10 min. Images of the stained plates were captured, and the colonies were counted. Colonies with a diameter of >60 μm were counted from three independent experiments.

### Cell migration

Cell migration was conducted as previously described by time-lapse imaging 21. Cells were seeded and cultured on collagen-coated plate in serum-free media. Time-lapse migration was traced using CCD video cameras (AxioCam MRm, Zeiss, Jena, Germany) and Track Point function of Image J software (NIH, Bethesda, MD, USA).

### Senescence-associated β-galactosidase (β-gal) staining

The cells were stained with the β-gal staining kit (#9860, CST) according to the manufacturer's instructions. Briefly, the cells were cultured on plates and exposed to staining reagents for 24 h at 37 °C in the absence of CO_2_, then washed with PBS before being observed under an inverted phase-contrast microscope (Olympus, Tokyo, Japan). The total number of cells and the number of blue-green senescent cells were counted in six random fields, and the percentage of senescent cells was calculated.

### Overexpression of four prognostic biomarkers in lung adenocarcinoma cells

Expression plasmids of pCMV3-SLC2A1-His (HG12102-CH), pCMV3-MTHFD2-Myc (HG16324-CM), and pCMV3-RRM2-Flag (HG18283-CF) were purchased from Sino Biological. A plasmid of pLNCX-HA-ADAM9 was constructed into the pLNCX vector as previously described 19. The plasmids were transiently transfected into the lung cancer cell lines using PolyJet™ *In Vitro* DNA Transfection Reagent (SL100688, SignaGen Laboratories) according to the manufacturer's instructions. Briefly, a total of 8 μg of plasmid mixture by mixing 2 μg of an individual plasmid with 6 μg of vector plasmids or plasmid combination of 4 genes (2 μg each gene) were used to over-express indicated proteins in lung cancer cells in 6 cm dishes. Protein levels were examined by western blot analysis.

### Tumor xenograft animal models

All the animal experiments were carried out in accordance with relevant guidelines and regulations at China Medical University, Taiwan, and were approved by the Institutional Animal Care and Use Committee of China Medical University, Taiwan. The SCID mice were purchased from the National Laboratory Animal Center (Taipei, Taiwan). In metastatic tumor animal models, control, individual gene, and 4 genes knockdown Bm7 lung cancer cells (5x10^4^ cells) were injected intracardially into 6-8 week-old SCID mice as previously described 22. Because Bm7 cells have stable luciferase expression, tumor metastasis in mice was detected by *in vivo* imaging system (IVIS) spectrum imaging system (Xenogen). Control and 4 gene knockdown A549 lung cancer cells (8x10^5^ cells) were intravenously injected into 6-8 week-old SCID mice (BioLASCO, Taiwan). Then the mice were kept in specific pathogen-free conditions, and the mouse survival was monitored.

### Human tissue array specimens

This research was approved by the Institutional Review Board at China Medical University Hospital (CMUH). Written informed consent was obtained from all patients prior to the study. Tissue specimens were identified using hospital medical records of lung cancer patients treated at CMUH from 2008 to 2013. According to the criteria of the World Health Organization (2004), stage I tumor specimens from LUAD patients who had not received chemotherapy or radiation treatment were included as comparators. In addition, formalin-fixed paraffin-embedded tissue blocks containing sufficient tumor material for sampling tissue cores were used. This resulted in 146 pairs of LUAD tissue and adjacent non-cancerous lung tissue being used for the tissue microarray. Another 25 tumor specimens from Stage 1B patients were included. We stratified the total 171 LUAD patients according to disease stage in 4 groups: (1) Stage 0 patients (N = 9); (2) Stage 1A patients (N = 97); (3) Stage 1B patients (N = 61); (4) Stage 2 to 4 (N = 4). The age of patients ranged from 26 to 85 years old at the point of surgery. After retrospective sample collection and analysis, the clinical outcomes of early-stage LUAD patients were determined using medical records.

### Immunohistochemical (IHC) staining

IHC staining was carried out as previously described 23. Briefly, tissue sections (4 μm) were dewaxed, dehydrated, and rehydrated. Then, citrate buffer was used for antigen retrieval, followed by 3% hydrogen peroxide to block endogenous peroxidase activity. After blocking the sections with 2.5% goat or horse sera, primary antibodies against ADAM9 (AF949, R&D Systems, MN, USA; at 1:1000 dilution), RRM2 (HPA056994, Sigma, MO, USA; at 1:300 dilution), MTHFD2 (H00010797-M01, Abnova, Taiwan; at 1:1500 dilution), and SLC2A1 (07-14011, Millipore, Billerica, MA, USA; at 1:3000 dilution) were added and incubated overnight at 4 °C. Specific staining was detected by goat anti-mouse or horse anti-rabbit horseradish peroxidase-conjugated secondary antibodies (Vector Laboratories), horseradish peroxidase-conjugated avidin-biotin complex from Vectastain Elite ABC Kit (Vector Laboratories), and AEC chromogen (Vector Laboratories). Counterstaining was performed using hematoxylin. The intensity score of each biomarker was assessed by pathologists according to the staining intensity from 0 to 3+ (0 = negative; 1 = weak; 2 = moderate; 3 = strong). The total IHC scores of the 4 biomarkers were summed up to measure the sum score. The sum score ranged from 1-3 was considered as a low IHC score group and 4-9 was as a high IHC score group.

### Statistical analysis

To compare the analyzed parameters between control and experimental groups, we used a two-tailed Student's *t-*test or two-way ANOVA for continuous variables. Statistical analysis was performed by GraphPad Prism Version 5.01 (GraphPad Software, San Diego, CA, USA). The log-rank test was used to determine survival differences, and a Cox proportional hazards regression model was used to quantitate the risk of patients with LUAD according to gene expression level. A *P* value <0.05 was considered statistically significant.

## Results

### Identification of prognostic candidates of LUAD

**Figure 1:**
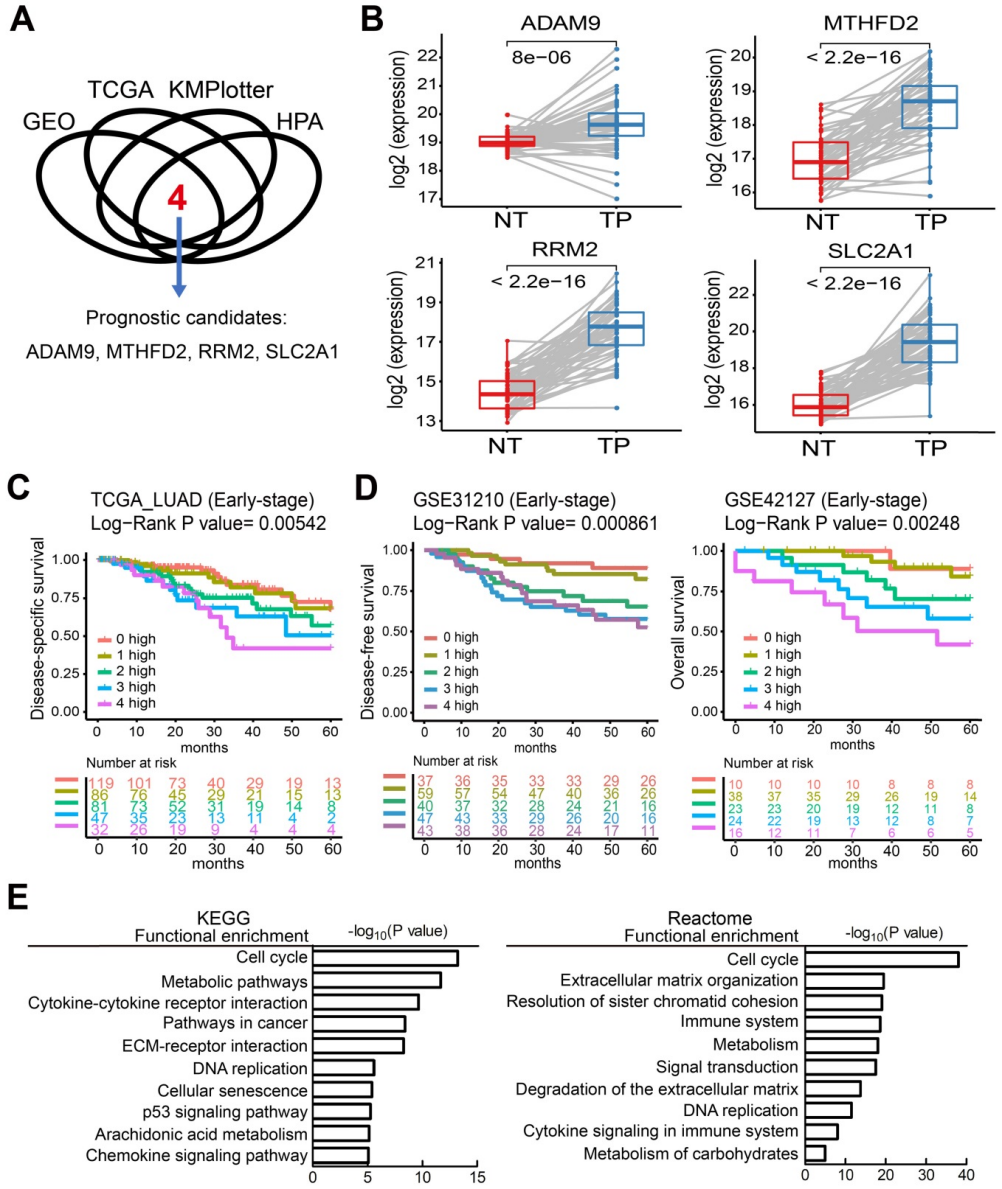
Four theranostic biomarkers identify LUAD patients with a poor prognosis. **(A)** The flowchart to identify the prognostic biomarkers of LUAD in public databases. **(B)** The RNA levels of the four genes from paired tumor-normal specimens from patients in the LUAD datasets of TCGA. NT, normal tissue; TP, tumor part. N = 59. *P* values are shown. **(C)** Kaplan-Meier survival curves of early-stage LUAD patients in TCGA datasets, grouped by different numbers of prognostic genes with high expression. 0 high: No genes with high expression. 1 to 4 high: the number of genes with high expression. HR: Hazard ratio. 0 high, HR reference. 1 high, HR = 1.18; 2 high, HR = 1.93; 3 high, HR = 2.65; 4 high, HR = 3.39. **(D)** Kaplan-Meier survival curves of early-stage LUAD patients in the GSE31210 (left) and GSE42127 (right) datasets. GSE42127 dataset only provided overall survival information. In GSE31210, 1 high (HR = 1.57); 2 high (HR = 3.61); 3 high (HR = 4.9); 4 high (HR = 4.95). In GSE42127, 1 high (HR = 1.31); 2 high (HR = 3.3); 3 high (HR = 5.25); 4 high (HR = 9.34). **(E)** Functional analysis of the differentially expressed genes in the 0 high and 4 high groups in the TCGA dataset.

To identify potential biomarkers for stratifying populations of LUAD patients by risk of metastatic disease with poor survival time, we included information from multiple independent datasets and selected the potential biomarkers with strict criteria (Figure 1A). Genes associated with patient survival independent of treatment received were considered an indicator of tumor aggressiveness, and only the genes that were related to prognosis by overexpression in tumors were selected. To increase the reliability of our method, we utilized several databases to screen potential biomarkers and took the results that were consistent across all of them. First, we identified the genes overexpressed in LUAD in two GEO datasets (GSE30219 and GSE31210) and the LUAD dataset of TCGA. Then, we chose the biomarker candidates with high staining in lung tumors but no or low detection in normal lung tissues according to the IHC staining in the Human Protein Atlas (HPA) database 24, which is a resource for pathology-based biomedical research. Next, we performed survival analysis in LUAD datasets from KMPlotter, as well as the two GEO datasets (GSE31210 and GSE30219) and the LUAD dataset from TCGA, to test whether their expression levels were related to survival (detail approach in methods). Finally, we identified 4 candidate genes—*ADAM9*, *MTHFD2*, *RRM2,* and *SLC2A1*—as potential prognostic biomarkers. In the paired normal and tumor tissue samples of the LUAD dataset in TCGA, the four genes were all overexpressed in primary tumors compared to the paired normal lung tissues (Figure 1B). Similar results were detected in both GEO datasets (GSE30219 and GSE31210, Figure S1A), suggesting that these four genes may be involved in tumorigenesis. Moreover, we used Causal Network Analysis, a component of Ingenuity® Pathway Analysis (IPA®) constructed from individual relationships derived from the literature, to investigate the causal relationships between 8 well-known LUAD driver genes and the four genes (Figure S2). We found that each of the four prognostic-related genes were linked to at least one LUAD driver gene, but there was no relationship between the four genes. These results indicate that *ADAM9*, *MTHFD2*, *RRM2,* and *SLC2A1* are likely involved in tumorigenesis.

### High expression of the four prognostic biomarkers correlates with high risk in early-stage LUAD patients

The unmet medical need for LUAD is to define 'high-risk patients' of early stage patients for adjuvant treatment. Thus, we investigated whether early-stage LUAD patients with higher expression of the four prognosis-related genes have reduced survival time. Using the TCGA dataset, we found that the survival time of early-stage LUAD patients with high expression of all four genes (4 high) had the shortest survival time, while those with low expression of all four genes (0 high) had the longest survival time (Figure 1C). Notably, the number of genes with high expression was directly related to the hazard ratio (HR) of death, as detected in the TCGA dataset (Figure 1C). Similar results were observed in LUAD patients of all stages (Figure S1B). Moreover, this phenomenon was validated in 4 independent LUAD cohorts: early stage in GSE31210 and GSE42127 (Figure 1D), all stages in GSE30219 and GSE8894 (Figure S1C). All four independent LUAD cohorts showed that an increased number of the four genes correlated with short survival time. We also performed multivariate survival analysis and utilized the concordance index (C-index) that ranges from 0.5 (no discriminating ability) to 1.0 (perfect ability to discriminate between cases with different outcomes) to evaluate the predictive ability of a survival model. By adjusting age, gender, pathologic stage, and smoking status as shown in Table 1, the multivariate result (P = 0.00032, C-index = 0.7221) is better than the univariate one (P = 0.00542, C-index = 0.6384), indicating the 4 biomarkers have an independent prognostic value.

To investigate which pathways are activated in association with the LUAD prognosis, we performed a functional analysis by comparing the differentially expressed genes in the 4 high group with the 0 high group in LUAD patients from the TCGA cohorts. This showed that several key pathways for cancer progression are active, including the cell cycle, metabolic pathways, DNA replication, and the immune system, by analysis in KEGG and reactome (Figure 1E).

Next, the predictive power of the four genes was evaluated in 32 different types of cancer in TCGA through the Customized Analysis function of the DriverDBv3 database 13. In 15 out of 32 (47%) cancer types, overexpression of a higher number of the four genes significantly correlated with shorter survival time (Table 2). This suggests that patients with high expression of all four genes have the worst outcome, and that reducing the expression of any one biomarker of the four may have survival benefits for cancer patients.

In general, the results of the bioinformatics analyses implicated that *ADAM9*, *MTHFD2*, *RRM2,* and *SLC2A1* are overexpressed in tumors and that the cumulative effect of their overexpression is associated with poor outcomes in multiple types of cancer. Thus, they may have potential to serve as prognostic biomarkers to identify the high-risk populations of LUAD and other cancer types.

### Knockdown of the four prognostic biomarkers suppresses cell proliferation and cell migration

**Figure 2:**
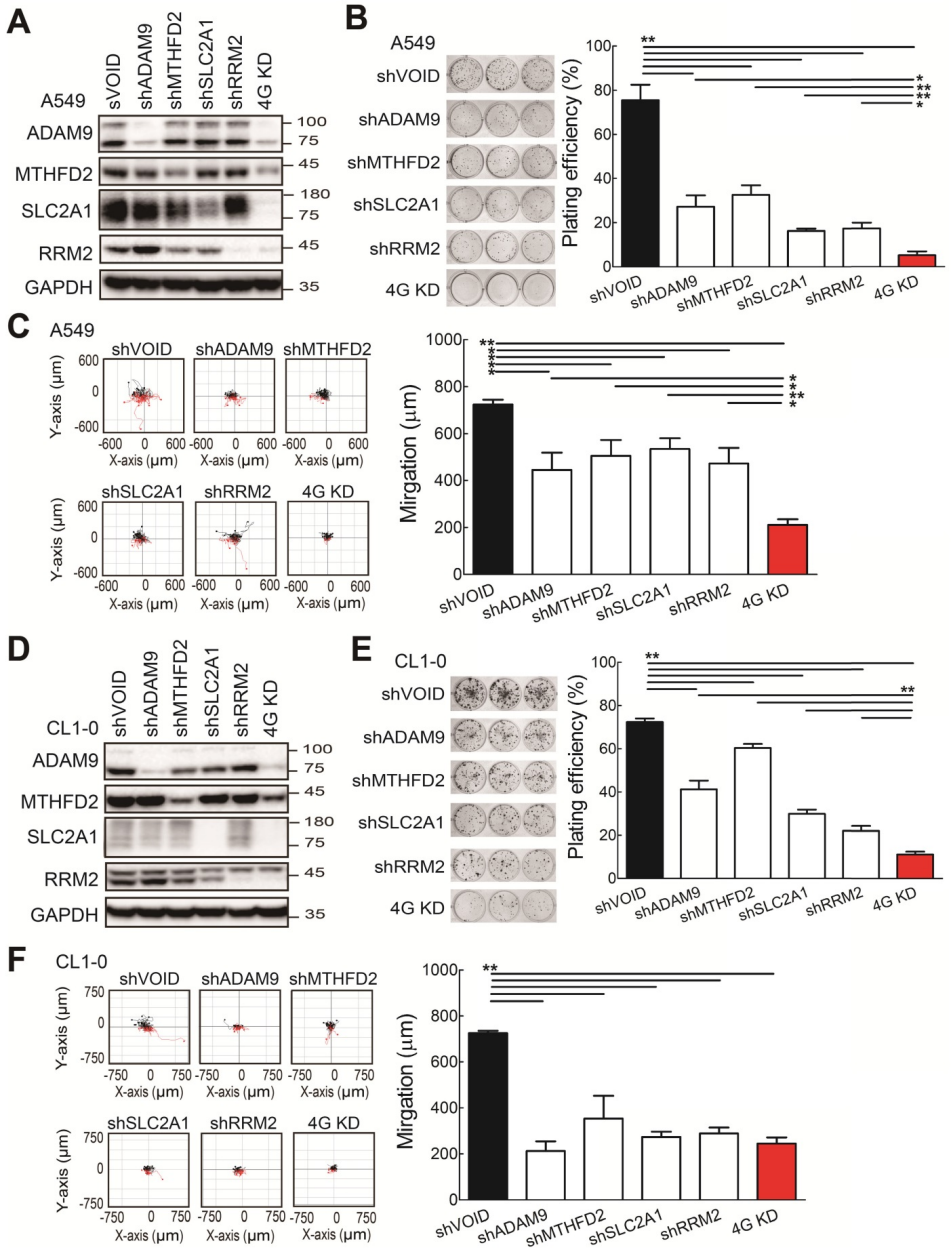
The functional assays of biomarker knockdown in A549 lung cancer cells using shRNAs against *ADAM9, MTHFD2*, *SLC2A1,* and *RRM2* individually or simultaneously (4G KD). **(A)** Western blots of control (shVOID), individual gene knockdown (shADAM9, shMTHFD2, shSLC2A1, and shRRM2), and 4G KD in A549 cells. **(B)** Colony formation of individual single gene knockdown and 4G KD A549 cells. Colonies were detected with crystal violet staining of the cell culture after 8 days (left) and quantified (right). **(C)** Cell migration ability of control, individual single gene knockdown, and 4G KD A549 cells. Cell migration was detected by time-lapse video microscopy (left) and quantified (right). **(D)** Western blots of control, individual gene knockdown, and 4G KD KD CL1-0 lung cancer cells. **(E)** Colony formation assay of control, individual gene knockdown, and 4G KD CL1-0 cells on day 11. **(F)** Cell migration ability of control, individual gene knockdown, and 4G KD CL1-0 cells. GAPDH served as the internal control. Error bars represent the mean ± SD of triplicate experiments. Statistical differences were analyzed using the unpaired *t* test (^*^P < 0.05, ^**^P < 0.01).

To investigate whether the four biomarker candidates contribute to LUAD progression, we analyzed their functions using a lentiviral shRNA system to knock down the individual genes (sh*ADAM9*, sh*MTHFD2*, sh*SLC2A1*, and sh*RRM2*) and all four genes simultaneously (4G KD) in A549 cells, a human LUAD line. Compared to control cells, individual gene knockdown of cells reduced the indicated protein expression and 4G KD reduced expression of all four proteins in A549 lung cancer cells (Figure 2A). The individual gene knockdown and 4G KD did show significantly suppressive effects on cell growth in colony formation assays and cell migration ability compared with control cells (Figure 2B-C). Notably, compared to the individual gene knockdown, 4G KD showed stronger effects on reducing cell growth and migration ability in A549 cells.

Consistent with the findings in A549 cells, the individual gene knockdown in CL1-0 lung cancer cells decreased the protein expression (Figure 2D), reduced the colony formation (Figure 2E) and cell migration ability (Figure 2F). Moreover, 4G KD showed reduced expression of all four proteins (Figure 2D) and caused greater effects on reducing plating efficiency in cell growth and migration ability compared with an individual knockdown in CL1-0 (Figure 2D-F). We also found similar observations in Bm7 lung cancer cells (Figure SF3). Thus, these findings indicate that reducing the expression of the four prognosis-related genes decreases lung cancer progression, and simultaneously reducing the levels of all 4 genes provides stronger effects on anti-cancer progression.

### Overexpression of the four prognostic biomarkers increases cell proliferation and cell migration

**Figure 3:**
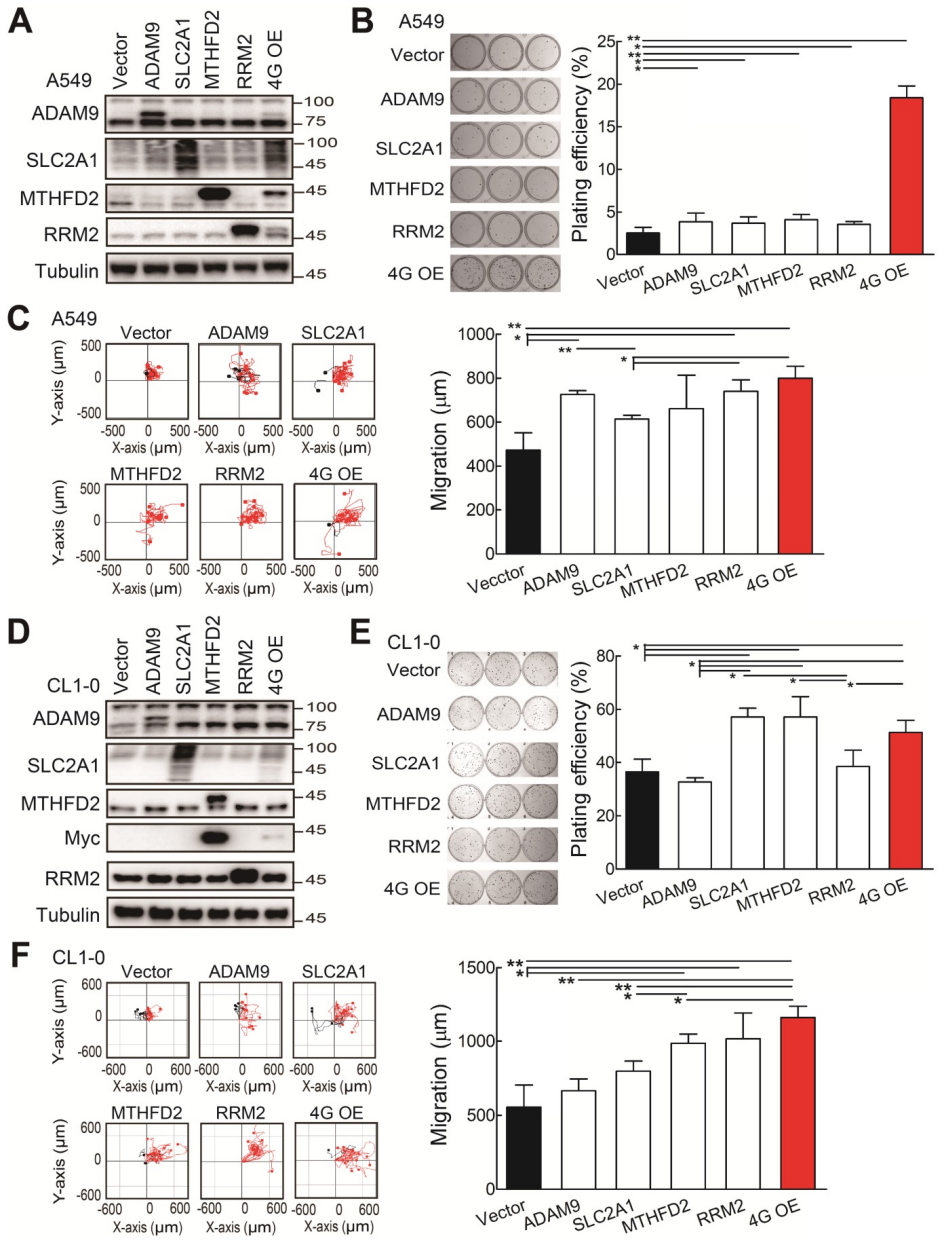
The functional assays of biomarker overexpression in A549 and CL1-0 cells using transiently transfection of plasmids expressing ADAM9, SLC2A1, MTHFD2, and RRM2 proteins individually or combined plasmids (4G OE). **(A)** Western blots of vector control, individual gene overexpression, and 4G OE in A549 cells. **(B)** Colony formation of control, individual single gene, and 4G OE A549 cells on day 8. **(C)** Cell migration ability of control, individual single gene, and 4G OE A549 cells. **(D)** Western blots of control, individual single gene, and 4G OE CL1-0 cells. **(E)** Colony formation assay of control, individual single gene, and 4G OE CL1-0 cells on day 8. **(F)** Cell migration ability of control, individual single gene, and 4G OE CL1-0 cells. Tubulin served as the internal control. Error bars represent the mean ± SD of triplicate experiments. Statistical differences were analyzed using the unpaired *t* test (^*^P < 0.05, ^**^P < 0.01).

Subsequently, we investigated whether the ectopic expression of all four biomarker candidates promoted LUAD progression. The individual gene or all 4 genes were transiently overexpressed in the A549 cells. Western blot analysis validated that the individual or 4 protein levels were elevated in A549 cells upon transfection with indicated expression plasmids (Figure 3A). The individual gene increased the plating efficiency in colony formation assays compared with the control group (Figure 3B). Notably, although the elevated levels of individual protein were lower in the 4 gene overexpression (4G OE) group compared to individual gene groups, 4G OE showed the highest cell growth effects (Figure 3B). In cell migration evaluation, most individual gene groups increased cell migration ability compared with control cells, and 4G OE showed a moderately higher migration effect than individual groups (Figure 3C). Similar results were observed for the overexpression of individual or 4 genes in the CL1-0 cells (Figure 3D). The individual gene or 4G OE showed enhanced effects on colony formation and cell migration ability compared with control cells (Figure 3E-F). Except the *RRM2* group, 4G OE demonstrated higher cell migration effects than individual gene groups (Figure 3F). We found the same phenomenon in Bm7 lung cancer cells that 4G OE significantly enhanced the cell growth and migration ability compared with control or individual gene groups (Figure S4). Thus, these data indicate that the four genes function together in promoting tumor progression.

### Knockdown of the four theranostic biomarkers induces cellular senescence

**Figure 4:**
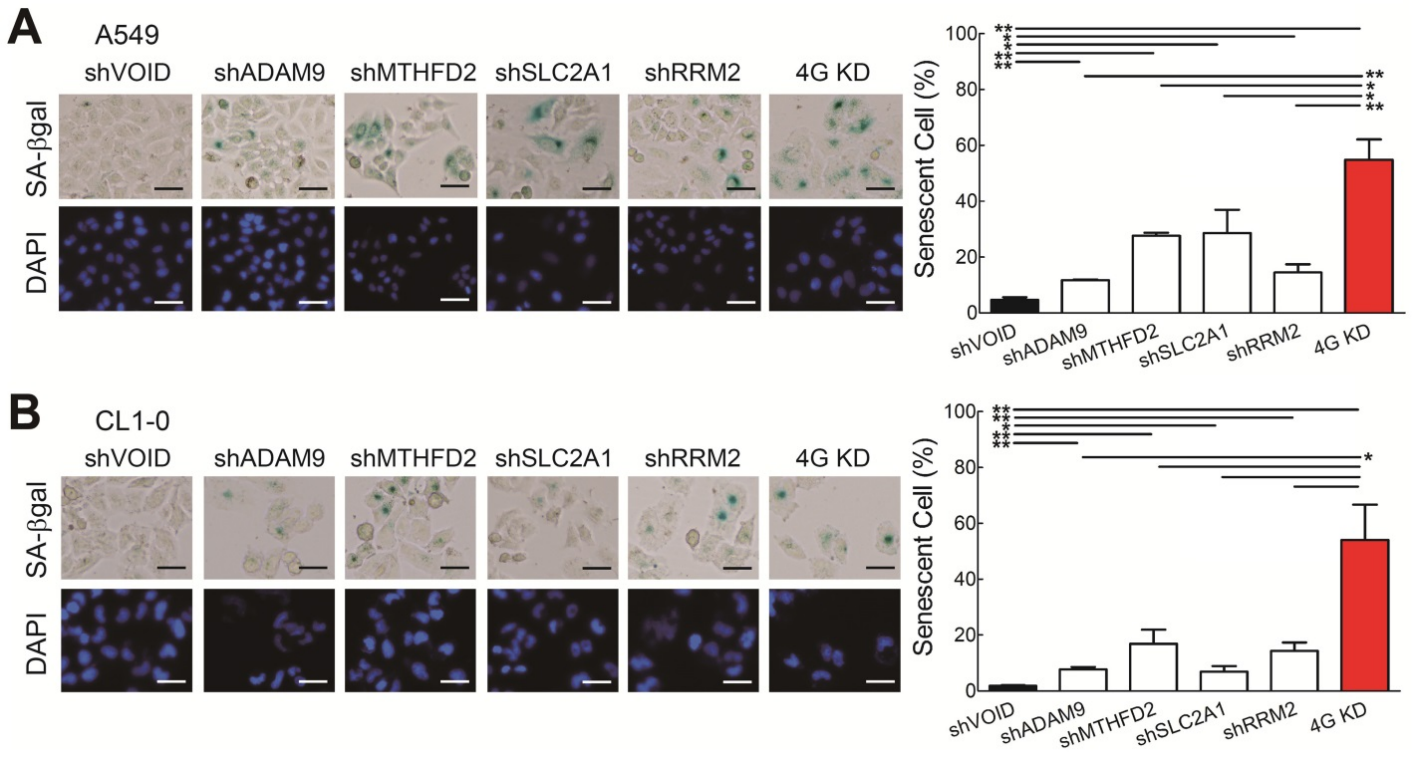
Knockdown of all 4 genes (4G KD) induces premature cellular senescence in lung cancer cells. **(A and B)** Representative images of SA-β-Gal-positive cells in control and 4G KD A549 cells (A) and CL1-0 cells (B). Cells were stained for SA-β-Gal and photographed under a phase-contrast microscope (left). Scale bar: 100 μm. Quantitative estimates of senescent cell fraction from microscope images (right) are the mean values ± SD. ^*^P < 0.05, **P < 0.01.

Next, we wanted to know which molecular mechanisms caused the 4G KD-mediated cell growth inhibition. Based on the functional analysis comparing the differentially expressed genes from the 4 high and 0 high groups in LUAD patients from TCGA (Figure 1E), cellular senescence is differentially regulated, suggesting that the 4G KD may result senescence. To validate it, we analyzed the senescence-specific marker, senescence-associated β-galactosidase (SA-β-Gal), and found that individual gene knockdown increased the SA-β-Gal staining cell percentage compared with control A549 cells and 4G KD cells had the strongest effects (Figure 4A). Similar results were detected in CL1-0 cells (Figure 4B). Taken together, this demonstrates that knocking down each one of the four genes induces cellular senescence and 4G KD provides the greatest senescence effect in LUAD.

### Knockdown of the four prognostic biomarkers in lung cancer cells reduces tumor malignancy and prolongs survival time *in vivo*

**Figure 5:**
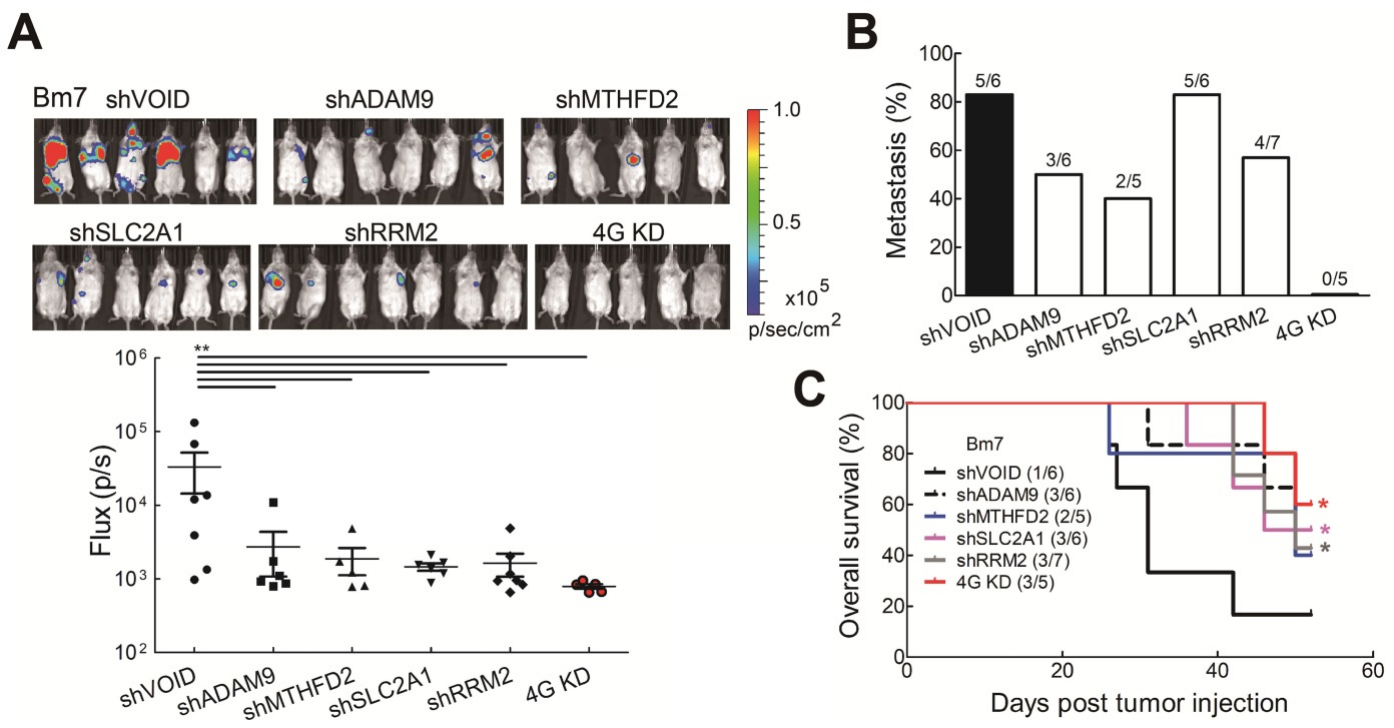
Knockdown of all 4 genes (4G KD) decreased tumor metastasis in lung tumor animal models. **(A)** Luciferase-expressing Bm7 control or gene-knockdown cells were intracardially injected into SCID mice. Representative images by IVIS from Day 15 post injection were shown (top). IVIS signals were quantified (bottom), mean ± SEM. **P < 0.01. **(B)** Tumor metastasis rate was calculated based on the IVIS images on Day 15. **(C)** Survival analysis of mice bearing Bm7 cancer cells. Parentheses indicate survived mice number/total number in each group on Day 52. Log-rank test. Comparison of each group to control group. *P < 0.05.

To further investigate whether reduced levels of the all four biomarkers influenced tumor malignancy *in vivo*, tumor metastatic ability was evaluated by intracardially injecting Bm7 cells with stable luciferase expression into SCID mice and tumor metastases were monitored by *in vivo* imaging system (IVIS) spectrum imaging system (Xenogen). The metastatic tumor signals were strongly reduced in individual gene knockdown and 4G KD groups on Day 15 (Figure 5A). Moreover, compared with individual gene knockdown groups, no tumor metastasis was detected in the 4G KD group on Day 15 (Figure 5B). The survival curve demonstrated that mice in the 4G KD group had a significantly longer overall survival time than the control group or some individual gene knockdown groups (Figure 5C). It demonstrates that the effect of 4G KD is superior to the knockdown of individual ones in reducing tumor metastasis *in vivo*. To further evaluate the effects of 4 genes in another metastatic tumor animal model, we transplanted control and 4G KD A549 cells by intravenous injection into SCID mice. The survival analysis indicated that mice inoculated with 4G KD A549 cells had a significantly longer overall survival time than the control group (Figure S6). It suggested that 4G KD is relative to tumor metastasis reduction. These results reveal that the 4 biomarkers have a significant connection with the progression of LUAD.

### The four prognostic biomarkers are associated with prognosis and clinicopathological tumor staging of early-stage LUAD

**Figure 6:**
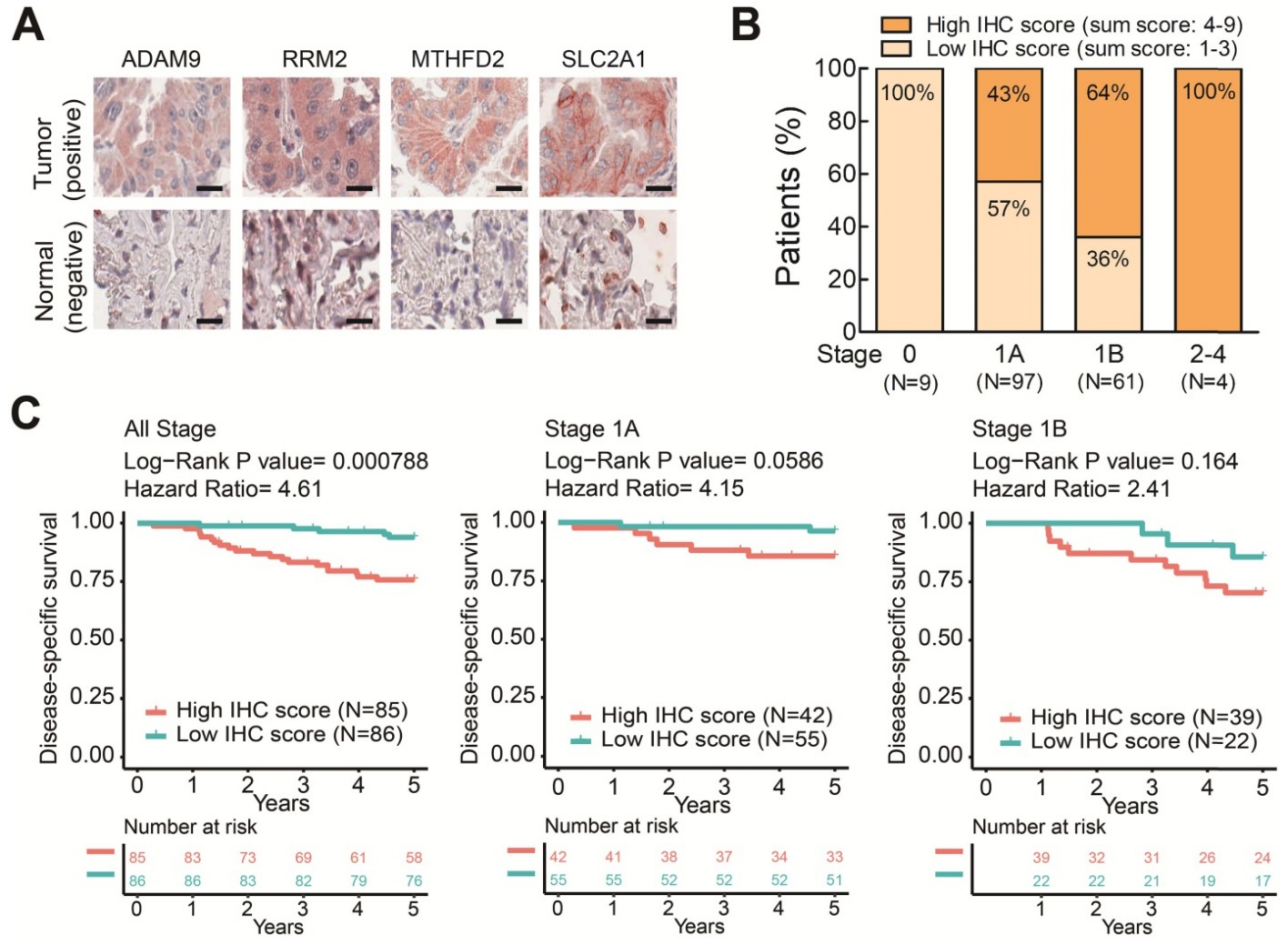
The protein levels of the four prognostic biomarkers in LUAD tissues correlates with clinicopathologic parameters. **(A)** IHC staining of individual biomarkers on the tissue array generated at CMUH. Scale bar is 25 μm. Images were captured using an Aperio CS Scanner with a 20X objective lens. Representative images show each biomarker with positive staining in tumors and negative staining in adjacent normal tissues. **(B)** The total IHC staining score of the four genes in 171 LUAD specimens was related to disease stage. The patients were divided into two groups according to their total IHC staining score: 1-3 (low IHC score) and 4-9 (high IHC score). **(C)** Kaplan-Meier survival curves of 171 LUAD patients (all stage) with low and high total IHC staining scores (left). Kaplan-Meier survival curves of stage 1A (middle) or 1B (right) LUAD patients with low and high total IHC staining scores.

To further elucidate the clinical relevance of these four theranostic biomarkers for LUAD, we examined the correlation between the four genes and clinicopathological tumor stage classification by IHC staining on LUAD tissue arrays. There were 171 pairs of tumor tissue and adjacent non-cancerous lung tissue obtained from patients treated at China Medical University Hospital (Table S2). We performed IHC staining on these LUAD tissue arrays using antibodies against the four biomarkers and observed major positive staining in tumors versus negative staining in adjacent normal lung tissues (Figure 6A and Figure S7). By summing the IHC scores of each protein and dividing the total scores into low (Sum IHC score = 1-3) and high (sum IHC score = 4-9) groups, we found the proportion of the high score group was elevated in the increased stages (Figure 6B). Moreover, the high score group had a shorter survival time than the low score group in these stage 1 patients (Figure 6C). Notably, in stage 1A patients, the high IHC score group had a significantly shorter survival time than the low score group (Figure 6C). A similar trend was observed in stage 1B patient although not reaching significant, probably due to the small sample size (Figure 6C). Taken together, these results demonstrated that the protein expression of the four genes detected by IHC was correlated with the LUAD stage and survival time. These findings provide the clinical evidence that high levels of the four biomarkers can identify populations of early-stage LUAD patients at high risk of mortality even the stage 1A patients who need no adjuvant treatment after surgery. The four prognosis-related genes not only serve as potential prognostic biomarkers of LUAD progression but also contribute to promoting the malignancy of LUAD in a synergic manner. Therefore, these four genes have strong potential to be used as theranostic biomarkers.

## Discussion

The current study identified 4 progression-related genes, *ADAM9*, *MTHFD2*, *RRM2,* and *SLC2A1,* based on an integrated bioinformatics approach. These genes were overexpressed in lung tumor tissues and may function as prognosis biomarkers. The number of these biomarkers with overexpression was correlated with poor survival of LUAD patients in five independent cohorts. Moreover, these genes contributed to indicators of lung cancer progression, including cancer cell growth and cell migration ability. Knocking down these four genes prolonged the survival time in a metastatic lung tumor mice model. These findings suggest that these four genes could serve as theranostic biomarkers to identify the high-risk population of early-stage LUAD patients who will benefit from receiving intensified therapy.

The uniqueness of this study is conducting an integrated bioinformatics approach including differential expression analysis, survival analysis, and protein expression to identify 4 promising prognostic genes. We demonstrated that the number of genes with high expression is related to hazard ratio (HR). To evaluate the effectiveness of our 4 biomarkers, we applied our analytic methods to other three previous studies which identifying 3 25, 16 26, and 18 27 prognostic signatures. As shown in Figure S8, the concordance index (C-index) of our 4 biomarkers is 0.6384, slightly better than the C-index of other 3 studies. Moreover, two studies, the 3- and 18- biomarkers, showed a small HR in 2 high (HR = 1.02) and 3-5 high (HR = 0.92) groups, respectively. We also demonstrated that the number of genes with high expression was correlated positively with high hazard ratio. Notably, although our 4 prognostic biomarkers were identified by gene expression datasets, they can be applied to clinical IHC staining for stratifying high-risk patients in early-stage LUAD.

The four theranostic biomarkers, *ADAM9*, *MTHFD2*, *RRM2,* and *SLC2A1*, have been reported to participate in tumorigenesis 18, 28-30. ADAM9, a metalloprotease, is thought to promote tumor progression through enhancing metastasis and angiogenesis 19. MTHFD2, a tetramethylfolate dehydrogenase enzyme involved in folate metabolism, is highly up-regulated to sustain cell proliferation 31. RRM2 is an enzyme with a role in DNA replication and repair 32. It could increase anti-apoptotic Bcl-2 protein expression and stability, which in turn promotes anti-apoptotic function 33. SLC2A1 (or glucose transporter 1, GLUT1), which transports glucose into cells, is required in glucose metabolism for fulfilling the high energy demands of cancer cells 25. The major functions of these four genes are involved in the hallmarks of cancer (Figure 1E), the essential biological processes that cancer cells rely on. These genes are likely to play a role in cancer cells' ability to adapt to environmental stress and pharmacological treatment. Their dysregulation likely leads to tumor progression and drug resistance. For example, SLC2A1 and dysregulated cellular metabolism is linked to drug resistance 34. Combination of SLC2A1 inhibitor and cisplatin or paclitaxel displayed synergistic therapeutic effects in lung and breast cancers 35. Despite the 4 biomarkers were identified independently from several datasets, we detected the moderate associations among the RNA expression of *MTHFD2*, *RRM2*, and *SLC2A1*, while *ADAM9* has weak correlations with other 3 genes (Figure S9). Moreover, protein levels of RRM2 were slightly reduced in either MTHFD2 or SLC2A1 knockdown lung cancer cells, but no alteration of MTHFD1 and SLC2A1 in RRM2 knockdown cells. It suggests that MTHFD2 or SLC2A1 may regulate RRM2 expression in a minor effect, probably through indirect regulation.

Senescence-associated cell-cycle arrest is a potential barrier to protect cells from transformation. Senescent cells typically exhibit a specific phenotype, the senescence-associated secretory phenotype (SASP), which consists of largely proinflammatory cytokines that participate in the clearance of cancer cells by attracting immune cells. However, accumulating senescent cells often drive cancer initiation and progression by a bystander effect in which SASP factors establish an immunosuppressive microenvironment and reinforce senescence of adjacent cells in a paracrine manner 36. In effect, early-stage senescence may protect cells from transformation, while prolonged senescence often promotes cancer development. In addition to telomere shortening in each cell division, senescence can be triggered by multiple genotoxic stresses, e.g., the epigenetic repression of the *INK4a*/*ARF* locus, DNA damage, and oxidative stress 37. Genotoxic chemotherapy and radiation can trigger stress-induced premature senescence in cancer cells during treatment, and this has been demonstrated to affect the therapeutic outcome 38. Accordingly, a strategy that first evokes senescence and then eliminates senescent cancer cells via the immune system or other mechanisms may provide anticancer benefits. Synolytic drugs, such as the natural product quercetin and the tyrosine kinase inhibitor dasatinib, which selectively induce apoptosis of senescent cells without affecting non-senescent cells, are being actively developed 39. In this study, we have demonstrated that reducing the levels of the four identified prognostic biomarkers in lung cancer cells can trigger cell senescence. By using them as theranostic biomarkers to predict prognosis and enhance therapeutic effects, synthetic lethal strategies targeting these biomarkers and then removing the senescent cells are necessary for an effective cancer remedy in the future.

The field of oncology has seen a paradigm shift in the treatment and molecular diagnosis of lung cancer in recent years, owing to the identification of mutations in driver genes. These breakthrough investigations provide a unique opportunity for selected lung cancer patients to receive targeted treatment options at the molecular level. The emergent therapeutic strategies have relied on specific biomarkers, which provide opportunities for a personalized approach to specific patient populations. Based on the development of screening techniques such as low-dose computed tomography, more and more lung cancer patients could be diagnosed at an early stage. The latest clinical guidelines suggest that high-risk early-stage LUAD patients should receive adjuvant treatment, but stratifying the high-risk population is still challenging. Although the heterogeneity of LUAD remains a key barrier to the investigation of novel biomarkers, the four theranostic genes identified in this study may serve as reliable biomarkers in LUAD patients for improving clinical management, treatment response, survival outcomes, and cost-effectiveness of drugs in the future.

## Supplementary Material

Supplementary figures and tables.

## Figures and Tables

**Table 1:** Multivariate adjustment of 4 genes in LUAD cancer in TCGA datasets

Condition	HR factor	Reference	Log-Rank P value of 5 years	HR of 5 years	n	C-index
Univariate	1 high	0 high	0.00542	1.18	365	0.6384
	2 high	0 high		1.93		
	3 high	0 high		2.65		
	4 high	0 high		3.39		
Multivariate (adjusted by age, gender, pathologic stage, and smoking status)	1 high	0 high	0.00032	1.48	338	0.7221
	2 high	0 high		1.94		
	3 high	0 high		2.85		
	4 high	0 high		3.04		

**Table 2:** Predictive power of 4 genes by the increase of gene number in 32 different types of cancer in TCGA datasets

Cancer type	Log-Rank P value*
**Kidney renal papillary cell carcinoma**	**0**
**Kidney renal clear cell carcinoma**	**9.17E-10**
**Uveal melanoma**	**5.56E-09**
**Kidney chromophobe**	**1.18E-08**
**Mesothelioma**	**0.000000108**
**Bladder urothelial carcinoma**	**0.0000053**
**Lung adenocarcinoma**	**0.0000651**
**Pancreatic adenocarcinoma**	**0.000103**
**Adrenocortical carcinoma**	**0.000481**
**Cholangiocarcinoma**	**0.00234**
**Lymphoid neoplasm diffuse large b cell lymphoma**	**0.00375**
**Liver hepatocellular carcinoma**	**0.00537**
**Cervical squamous cell carcinoma**	**0.0129**
**Low grade glioma**	**0.021**
**Breast carcinoma**	**0.0265**
Colon adenocarcinoma	0.0757
Prostate adenocarcinoma	0.111
Uterine corpus endometrial carcinoma	0.113
Glioblastoma multiforme	0.237
Uterine carcinosarcoma	0.332
Thymoma	0.367
Thyroid carcinoma	0.378
Head and neck squamous cell carcinoma	0.383
Sarcoma	0.384
Testicular germ cell tumors	0.445
Esophageal carcinoma	0.513
Skin cutaneous melanoma	0.536
Pheochromocytoma and paraganglioma	0.546
Ovarian serous cystadenocarcinoma	0.563
Rectum adenocarcinoma	0.731
Lung squamous cell carcinoma	0.779
Stomach adenocarcinoma	0.866

Statistical differences were analyzed using the unpaired t test. *P* values <0.05 are shown in bold. *Kaplan-Meier survival analysis of cancer patients, grouped by different numbers of prognostic genes with high expression.

## Abbreviations

ADAM9: a disintegrin and metalloproteinase 9
EGFR: epidermal growth factor receptor
FBS: fetal bovine serum
HPA: Human Protein Atlas
GFP: green fluorescent protein
HR: hazard ratio
IHC: Immunohistochemical
LUAD: lung adenocarcinoma
MTHFD2: methylenetetrahydrofolate dehydrogenase
NCCN: National Comprehensive Cancer Network
NSCLC: non-small-cell lung cancer
RRM2: Ribonucleoside-diphosphate reductase subunit M2
SA-β-Gal: senescence-associated-β-galactosidase
SASP: senescence-associated secretory phenotype
shRNA: short-hairpin RNA
SLC2A1: solute carrier family 2 member 1
TCGA: The Cancer Genome Atlas
