# Supplementary material for: Identification of theranostic factors for patients developing metastasis after surgery for early-stage lung adenocarcinoma
Source: Theranostics. 2021 Jan 26;11(8):3661–75. doi: 10.7150/thno.53176 (PMC7914355; doi:10.7150/thno.53176)
Supplement: Supplementary file 1 — Supplementary figures and tables. [file thnov11p3661s1.pdf]

Supplemental Information

Identification of theranostic factors for patients developing metastasis after surgery for early-stage lung adenocarcinoma

Includes:

1. Supplementary Table
2. Supplementary Figures

Supplementary Table

Table S1. Four individual short hairpin RNA sequences.

gene name	Oligo Sequence	Target
shADAM9	CCGGCCCAGAGAAGTTCCTATATATCTCGAGATATATAGGAACTTCTCTGGGTTTTTG	CCCAGAGAAGTTCCTATATAT
shRRM2	CCGGCGGAGGAGAGAGTAAGAGAAACTCGAGTTTCTCTTACTCTCTCCTCCGTTTTTG	CGGAGGAGAGAGTAAGAGAAA
shMTHFD2	CCGGCGAATGTGTTTGGATCAGTATCTCGAGATACTGATCCAAACACATTCGTTTTTG	CGAATGTGTTTGGATCAGTAT
shSLC2A1	CCGGGCCACACTATTACCATGAGAACTCGAGTTCTCATGGTAATAGTGTGGCTTTTTG	GCCACACTATTACCATGAGAA

Table S2. The demographic table of CMUH cohort (171 lung cancer patients)

Variable	High IHC score	Low IHC score	P value
Gender n (%)			
Male	49 (57.65)	31 (36.05)	0.006
Female	36 (42.35)	55 (63.95)	
Smoking n (%)			
No	41 (59.42)	60 (82.19)	0.003
Yes	28 (40.58)	13 (17.81)	
Age Mean ± SD	63.49 ± 11.46	58.58 ± 13.42	0.011
pTNM_stage n (%)			
0	0 (0.00)	9 (10.47)	< 0.00001
1A	42 (49.41)	55 (63.95)	
1B	39 (45.88)	22 (25.58)	
2-4	4 (4.71)	0 (0.00)	

Supplementary Figures

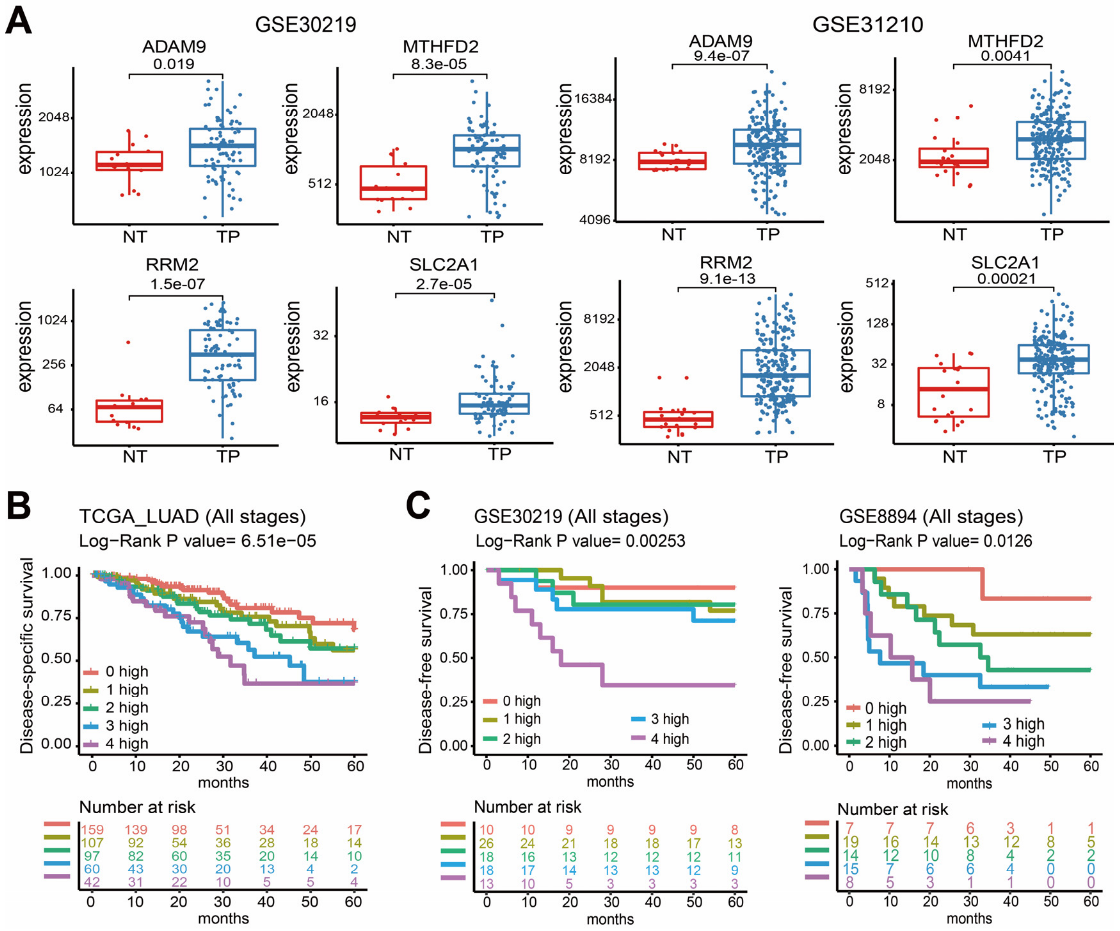

Figure S1, related to Figure 1. (A) The RNA levels of the four genes from normal and tumor specimens in the LUAD datasets of GSE30219 (left) and GSE31210 (right). NT, normal tissue; TP, tumor part. GSE30219, NT = 14; TP = 85. GSE31210, NT = 20; TP = 226. (B) Kaplan-Meier survival curves of LUAD patient in all stages with grouping in different numbers of the genes with high expression in TCGA datasets. HR: Hazard ratio. 0 high, HR reference. 1 high, HR = 1.61; 2 high, HR = 1.79; 3 high, HR = 3.12; 4 high, HR = 3.73. (C) Kaplan-Meier survival curves of LUAD patients with all stages in the GSE30219 and GSE8894 datasets. In GSE30219, 1 high (HR = 2.13); 2 high (HR = 2.03); 3 high (HR = 3.15); 4 high (HR = 10.8). In GSE8894, 1 high (HR = 2.99); 2 high (HR = 4.9); 3 high (HR = 8.87); 4 high (HR = 11.28).

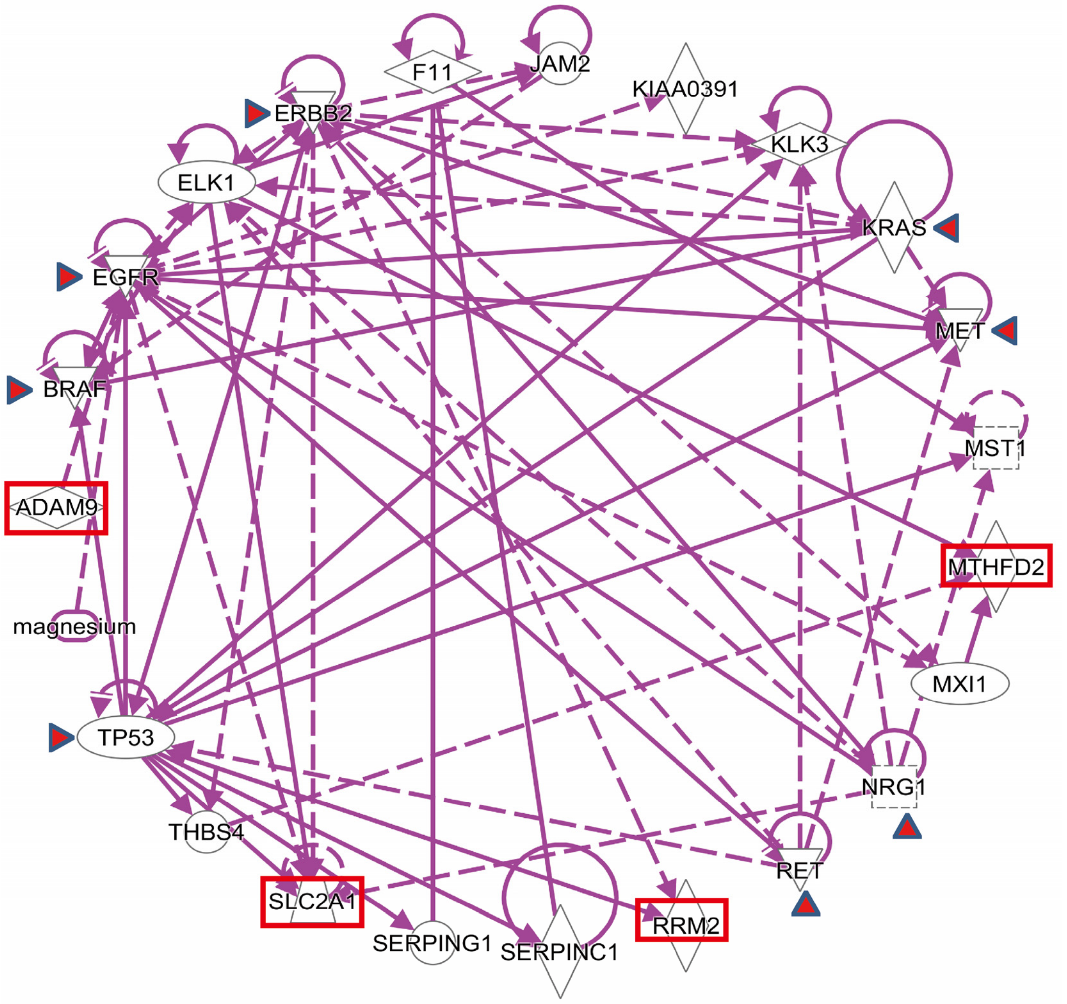

Figure S2. The link of 4 prognosis-related genes and drivers-network of LUAD dataset by Ingenuity® Pathway Analysis (IPA®). Red arrowhead, driver genes. Red rectangle, 4 prognostic biomarkers.

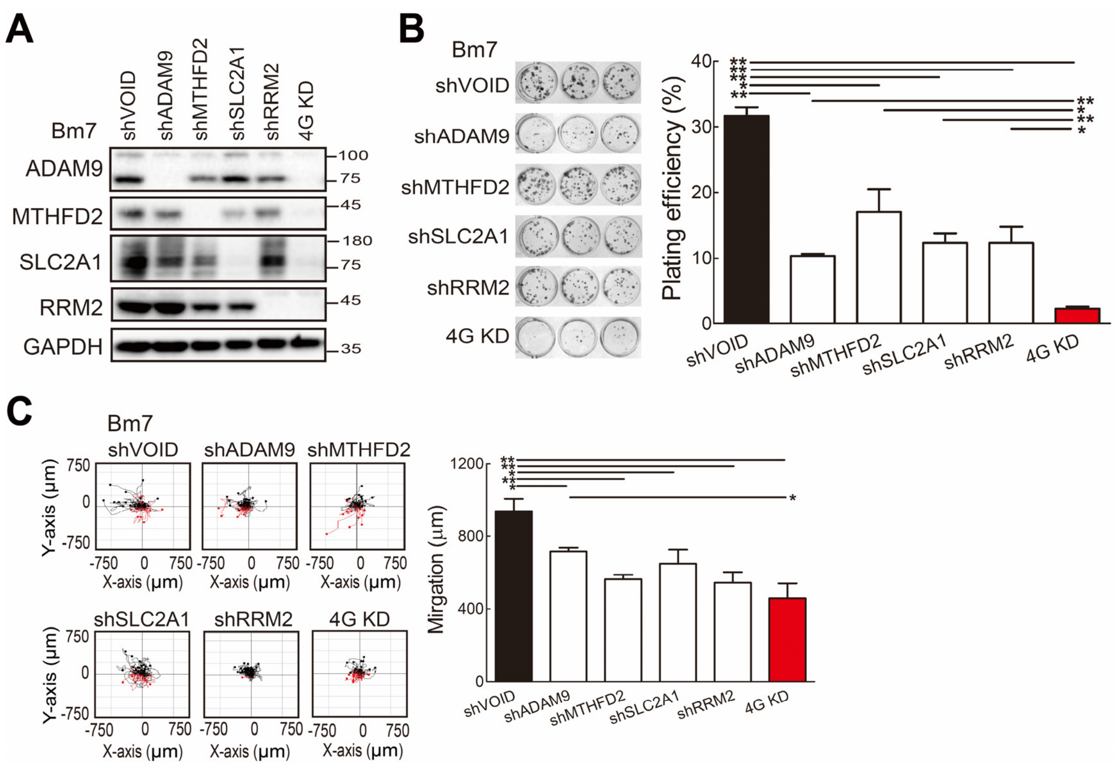

Figure S3, related to Figure 2. The biomarker knockdown in Bm7 cells using shRNAs targeted to *ADAM9, MTHFD2*, *SLC2A1,* and *RRM2* individually or combined (4G KD) reduced cell migration ability. (A) Western blots of control (shVOID), individual gene knockdown (shADAM9, shMTHFD2, shSLC2A1, and shRRM2), or 4G KD in Bm7 cells. (B) Colony formation assay of control, individual gene knockdown, and 4G KD Bm7 cells on day 8. (C) Cell migration ability of individual single gene knockdown cells. Error bars represent the mean ± SD of triplicate experiments. Statistical differences were analyzed using the unpaired *t* test ($^{*}P < 0.05$, $^{**}P < 0.01$).

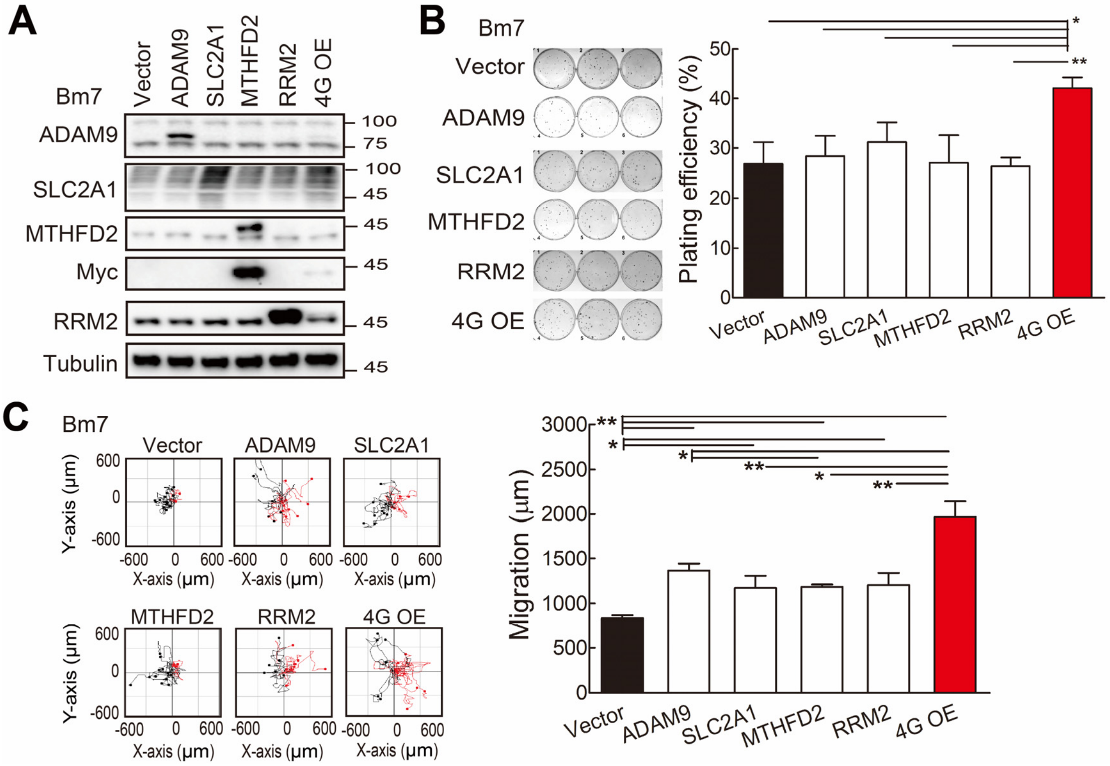

Figure S4, related to Figure 3. The functional assays of biomarker overexpression in Bm7 cells using transiently transfection of plasmids expressing ADAM9, SLC2A1, MTHFD2, and RRM2 proteins individually or combined plasmids (4G OE). (A) Western blots of vector control, individual gene overexpression, and 4G OE in Bm7 cells. (B) Colony formation of control, individual single gene, and 4G OE Bm7 cells on day 8. (C) Cell migration ability of control, individual single gene, and 4G OE Bm7 cells. Error bars represent the mean ± SD of triplicate experiments. Statistical differences were analyzed using the unpaired *t* test ($^{*}P < 0.05$, $^{**}P < 0.01$).

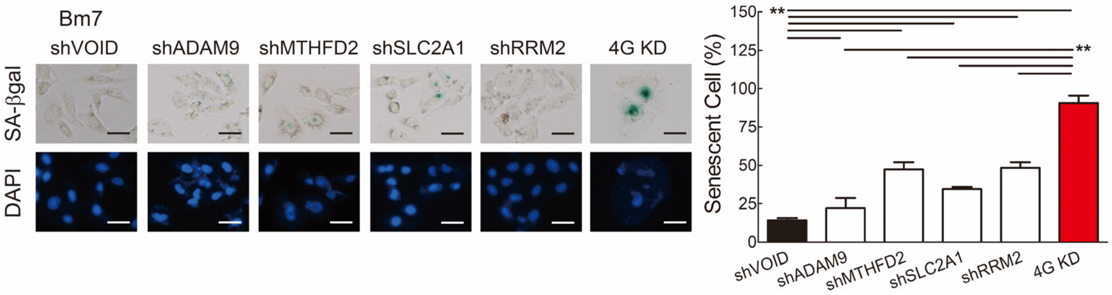

Figure S5, related to Figure 4. Knockdown of all 4 genes (4G KD) induces premature cellular senescence in Bm7 lung cancer cells. Senescent cells were stained for SA-β-Gal and photographed under a phase-contrast microscope (left). Scale bar: 100 µm. Quantitative estimates of senescent cell fraction from microscope images (right) are the mean values ± SD. ***P*<0.01.

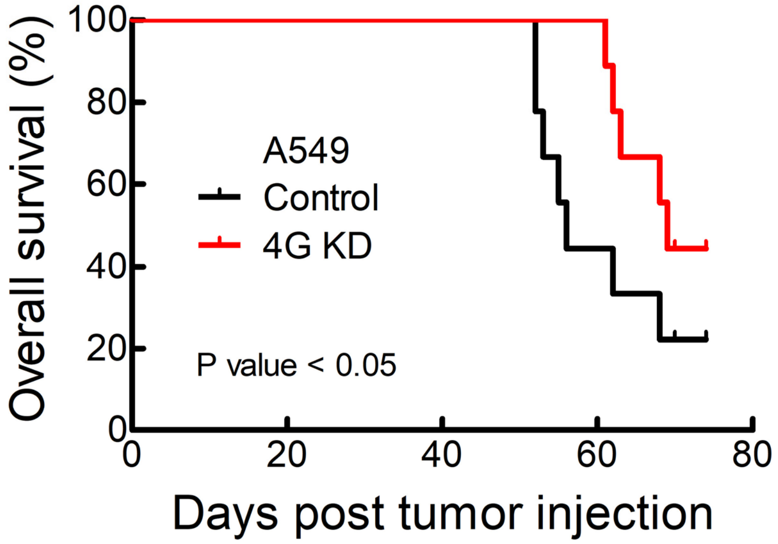

Figure S6, related to Figure 5. Survival analysis of mice bearing A549 cancer cells. Control or 4G KD A549 cancer cells were intravenously injected into SCID mice. Mouse survival time was monitored for 74 days. Control group (N = 9); 4G KD group (N = 9).

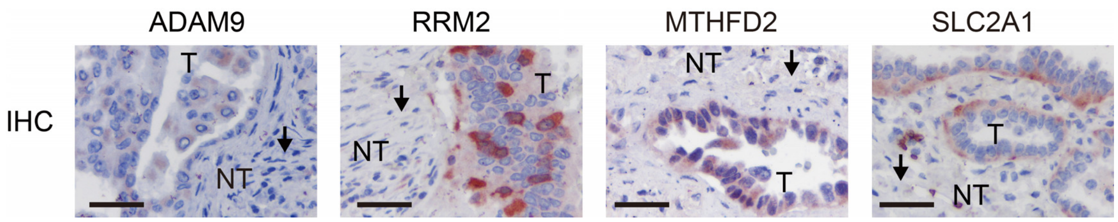

Figure S7, related to Figure 6. IHC staining of individual biomarkers on the slides of formalin-fixed paraffin-embedded tissue blocks of stage 1B lung cancer patients from CMUH. An arrow indicates the negative staining in the adjacent non-tumoral tissue. The positive staining in the tumor (T) and the negative staining in adjacent non-tumoral (NT) tissues. Scale bar is 50 μm.

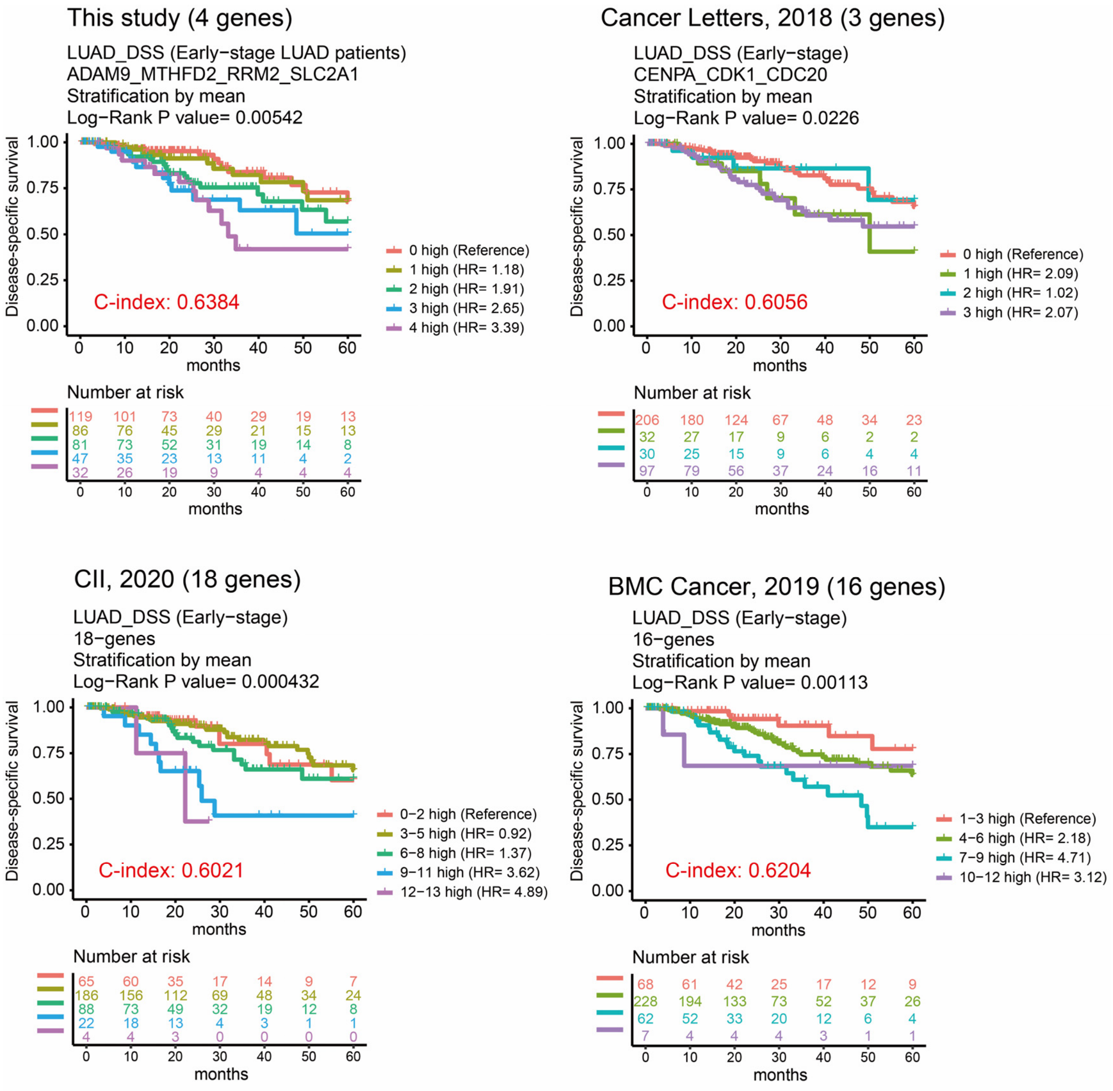

Figure S8. Comparison of combined 4 diagnostic biomarkers with other reported biomarkers. Four biomarkers in this study; 3 biomarkers in Cancer Letter, 2018 (PMID: 29608985); 18 biomarkers in Cancer Immunol Immunother, 2020 (PMID: 32372138); 16 biomarkers in BMC Cancer, 2019 (PMID: 31488089). These biomarkers were used to compare the Kaplan-Meier survival curves and concordance index (C-index) of early stage LUAD patients in TCGA datasets, grouped by different numbers of prognostic genes with high expression.

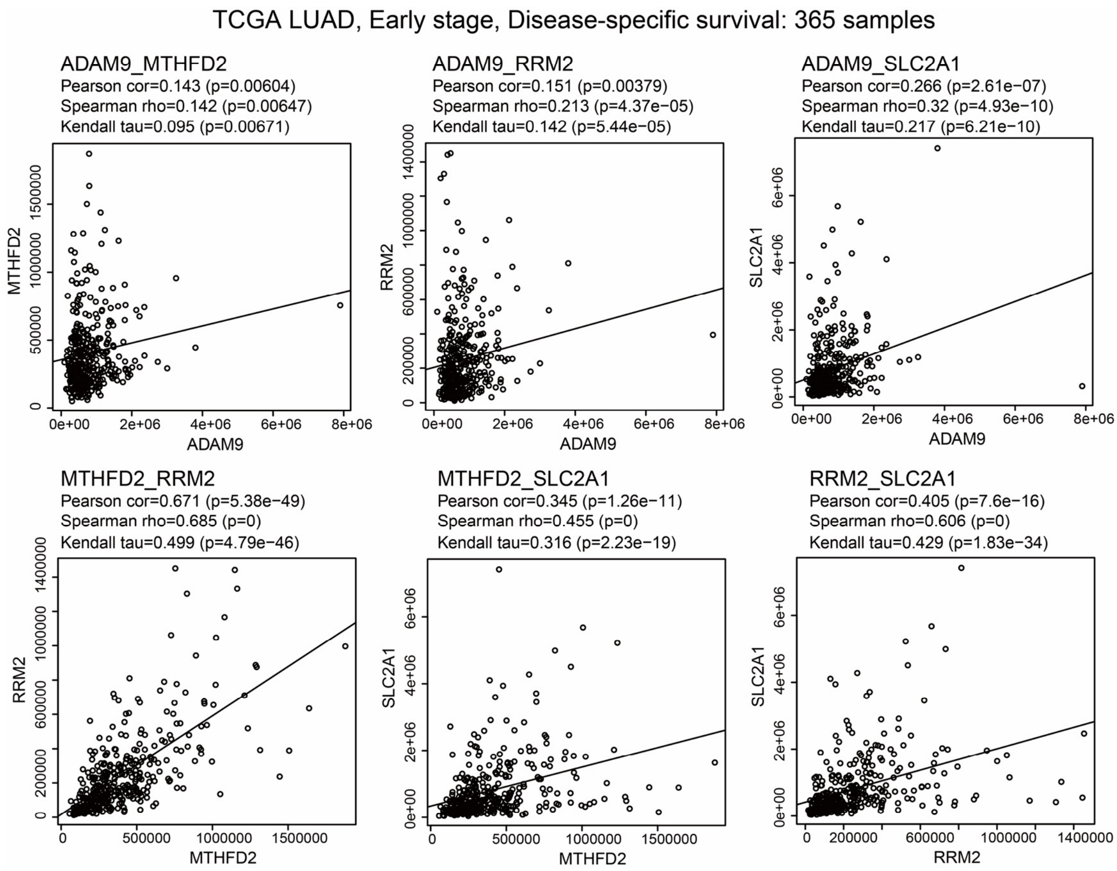

Figure S9. Correlation analysis of the 4 genes in early stage LUAD patients in TCGA datasets.
